# Pemetrexed sensitizes cisplatin therapy by inducing ferroptosis in NSCLC cells

**DOI:** 10.3389/fphar.2025.1764937

**Published:** 2026-01-21

**Authors:** Yumin Wang, Xin Zhang, Yuwei Cao, Ge Zhang, Yonglin Zhu, Yulin Li, Jichao Chen

**Affiliations:** 1 Department of Respiratory and Critical Care Medicine, Aerospace Center Hospital, Peking University Aerospace School of Clinical Medicine, Beijing, China; 2 Department of General Ward, Aerospace Center Hospital, Peking University Aerospace School of Clinical Medicine, Beijing, China

**Keywords:** cisplatin, drug resistance, ferroptosis, non-small-cell lung cancer, pemetrexed

## Abstract

**Background:**

Cisplatin (DDP) is the first-in-class drug for advanced and non-targetable non-small-cell lung cancer (NSCLC). Platinum-based chemotherapy combined with pemetrexed (PEM) is frequently recommended as the first-line therapeutic regimen for NSCLC. However, the mechanisms of how PEM boosts the antitumor activity of DDP are largely unknown. Emerging evidence indicated that DDP could induce ferroptosis, a new type of regulated cell death (RCD) characterized by iron-dependent toxic build-up of lipid peroxides on cellular membranes. It is tempting to speculate whether PEM increases the sensitivity of NSCLC to DDP through inducing ferroptosis.

**Methods:**

In the present study, we first used RNA-seq and KEGG analysis to examine differentially expressed genes in PEM-challenged NSCLC cells. The effect of PEM on increased DDP-mediated anticancer activity was examined via a cytotoxicity assay and Western blot. PEM-triggered ferroptosis in DDP-treated NSCLC was observed via a lipid peroxidation assay, a labile iron pool assay, and a Western blot in the presence or absence of ferroptosis inhibitors.

**Results:**

In the present study, we found that the ferroptosis-related pathway was enriched by PEM. PEM significantly enhanced the ability of cisplatin to inhibit cell viability and proliferation in NSCLC cells. The combination of PEM and DDP synergistically induced ferroptosis, as evidenced by the increased reactive oxygen species (ROS), lipid peroxidation, and Fe^2+^ and decreased SOD. PEM facilitated DDP-mediated upregulated expression of pro-ferroptosis proteins (ACSL4, 12LOX, COX2, DMT1, TFR1, and TF) and downregulated the expression of anti-ferroptosis proteins (SLC7A11, GPX4, FPN1, FTH1, FTL, DHODH, FSP1, and GCH1). However, the effects were reversed by ferroptosis inhibitor ferrostatin-1 or deferoxamine in NSCLC cells.

**Conclusion:**

In summary, these results provide *in vitro* experimental evidence that PEM boosts the antitumor activity and increases the sensitivity of NSCLC cells to DDP by inducing ferroptosis.

## 1 Introduction

Lung cancer remains a leading cause of cancer-related mortality worldwide (Sung et al., 2021). It is broadly classified into two types: small-cell lung cancer (SCLC) and non-small-cell lung cancer (NSCLC). NSCLC represents the major histological subtype, accounting for more than 80% of all lung cancers (Rotow and Bivona, 2017; Leiter et al., 2023; Siegel et al., 2023; LoPiccolo et al., 2024; Otano et al., 2023). It primarily includes lung squamous cell carcinoma (LUSC) and lung adenocarcinoma (LUAD). Globally, the incidence of lung cancer is estimated to increase significantly in the coming decades (Hirsch et al., 2017; Soerjomataram and Bray, 2021).

Despite advances in treatment, the 5-year overall survival for lung cancer patients remains low, ranging from 4% to 17%, depending on disease stage and geographical factors (Hirsch et al., 2017). Current therapeutic strategies for NSCLC include surgery, targeted therapy, immunotherapy, and radiotherapy. For patients with advanced NSCLC, platinum-based chemotherapy remains the standard first-line regimen (Nagasaka and Gadgeel, 2018). This is often combined with agents such as pemetrexed, vinorelbine/taxanes, or gemcitabine. However, the efficacy of platinum-based chemotherapy is limited by chemoresistance, which varies considerably among individuals (Yin et al., 2016; Wang et al., 2024). Therefore, there is a clear need to explore novel mechanisms and therapeutic strategies to improve outcomes in NSCLC (Herbst et al., 2018; Lim and Ma, 2019).

Ferroptosis is a new type of regulated cell death (RCD) characterized by iron-dependent toxic build-up of lipid peroxides on cellular membranes (Lei et al., 2022; Kobayashi et al., 2023; Yunchu et al., 2023; Zhu et al., 2025). Conventional therapies that include chemotherapy, radiotherapy, immunotherapy, and targeted cancer therapies exert antitumor activity by inducing ferroptosis (Sun et al., 2016; Guo et al., 2018; Wang et al., 2019; Lei et al., 2020; 2022). Therefore, ferroptosis functions as a novel strategy for cancer therapy, and ferroptosis targeting even offers a therapeutic strategy for cancers that are resistant to conventional therapies, including NSCLC (Wang et al., 2023a, 2023b; Wang et al., 2024). Therefore, uncovering the molecular complexities underlying ferroptosis regulation in NSCLC may create more effective therapeutic strategies for NSCLC.

Pemetrexed (PEM), a folate analog metabolic inhibitor for mammalian cells, is toxic to several cancer cells by interfering with their biosynthesis of nucleotides (Vora et al., 2021). Previous studies have shown the synergistic antitumor effect of pemetrexed in combination with cisplatin (DDP) in the treatment of NSCLC (Yu et al., 2022). Given that DDP induces ferroptosis in various cancers, the aim of the present study is to investigate whether PEM could induce ferroptosis and increase the sensitivity of NSCLC cancer cells to DDP therapy by inducing ferroptosis, and also elucidate the detailed mechanisms underlying how PEM boosts the antitumor activity of DDP in NSCLC cells. In the present study, we uncover a novel mechanism underlying how PEM kills NSCLC cells by inducing ferroptosis.

## 2 Materials and methods

### 2.1 Materials

The human NSCLC A549 and H1299 cell lines were obtained from the China Center for Type Culture Collection (CCTCC). Complete protease inhibitor was obtained from Roche Diagnostics GmbH (Penzberg, Germany). Modified Dulbecco’s Eagle’s medium supplement was obtained from Gibco Invitrogen Corporation. Antibodies and other reagents used in the present study are shown in Supplementary Table S1. All other chemicals used were of commercially available high grades.

### 2.2 Cell culture and treatment

A549 cells were cultured in RPMI-1640 medium, while H1299 cells were maintained in Dulbecco’s modified Eagle’s medium (DMEM) (Zhang et al., 2025). Both media were supplemented with 10% fetal bovine serum (FBS), 100 μg/mL streptomycin, and 100 U/mL penicillin. Cells were incubated at 37 °C in a humidified atmosphere containing 5% CO_2_. All cell lines were confirmed to be free of *Mycoplasma* contamination.

### 2.3 Determination of cell viability

The cytotoxicity of pemetrexed (PEM) and DDP in A549 and H1299 cells was evaluated by the CCK8 Assay Kit. Cells were seeded in a 96-well culture plate at a density of 0.5 × 10^3^ cells per well in 100 μL medium overnight. Then, different concentrations of PEM or DDP (100 nM for PEM or 200 nM for DDP), as well as PEM (100 nM) and DDP (200 nM), were added and incubated for 72 h, respectively. For the rescue assay, PEM and DDP were co-treated with pharmacological ferroptosis inhibitors of ferrostatin-1 (Fer-1, 1 µM) and deferoxamine (DFO, 100 µM) for 24 h. After treatment, 10 μL CCK-8 was added to each well, and incubation continued for 2 h at 37 °C.

### 2.4 Intracellular reactive oxygen species (ROS) generation detection

Intracellular ROS fluorescence assay kits (WLA131; Wanleibio, China) were used to measure the levels of ROS according to the kit instructions.

### 2.5 Determination of intracellular iron levels

Intracellular iron levels in the cell supernatants were examined with an Iron Assay Kit (cat. no. E-BC-K881-M; Elabscience, China). The concentration of iron ions in the cells was determined at 520 nm using a microplate reader. The intracellular iron ion levels were then normalized by cell numbers.

### 2.6 GSH, MDA, and SOD analyses

Logarithmically grown A549 and H1299 cells were inoculated into 6-well plate dishes at a density of 3 × 10^5^ cells/mL, with 5 mL per dish. The cells were next treated for 72 h with PEM (100 nM), or DDP (200 nM for DDP), or PEM (100 nM) plus DDP (200 nM for DDP) in the presence or absence of Fer-1 (1 µM) and DFO (100 µM). After washing with PBS, the cells were homogenized by ultrasonic crushing to obtain the cell supernatants after centrifugation. The levels of malondialdehyde (MDA) (WLA048; Wanleibio, China), glutathione (GSH) activity (WLA105; Wanleibio, China), and SOD (WLA110; Wanleibio, China) were measured at 405 nm, 450 nm, and 530 nm, following the manufacturers’ instructions.

### 2.7 Western blot analysis

Changes in the expression levels of the indicated proteins were assessed using Western blotting, as described previously (Li et al., 2022). A549 cells were washed twice with cold PBS and lysed in RIPA buffer supplemented with a complete phosphatase and protease inhibitor cocktail. Protein quantification was performed using the BCA assay kit. Proteins (20 µL) were separated via SDS-PAGE and transferred to a PVDF membrane. The membranes were blocked with 5% skim milk for 1 h at room temperature, and then incubated with the following primary antibodies overnight at 4 °C with the appropriate primary antibodies. Appropriate secondary antibodies conjugated to horseradish peroxidase were used for 2 h at room temperature. The blots were developed using an ECL Western blotting detection reagent (Santa Cruz Biotechnology, United States).

### 2.8 RNA sequencing (RNA-seq)

Total RNA was extracted from A549 cells that were simulated with or without PEM (100 nM) using TRIzol reagent (15596026, Invitrogen, United States). Then, a total of six samples were sent to the BoAo Corporation (China) for further RNA-seq detection and analysis. Differentially expressed genes (DEGs) between the two groups were identified by the DEGseq method. The pathway analysis for DEGs was performed based on the KEGG database. Detailed methods are shown in the Supplementary Material.

### 2.9 Statistical analysis

All data are presented as the mean ± SEM. Data were subjected to statistical analysis using one-way analysis of variance (ANOVA) followed by Student’s t-test using GraphPad Prism 6.0 software (GraphPad Software, Inc., San Diego, CA, United States). Mean values were considered to be statistically significant at *P* < 0.05.

## 3 Results

### 3.1 Pemetrexed sensitizes cisplatin-induced cytotoxic effects in NSCLC cells

**FIGURE 1:**
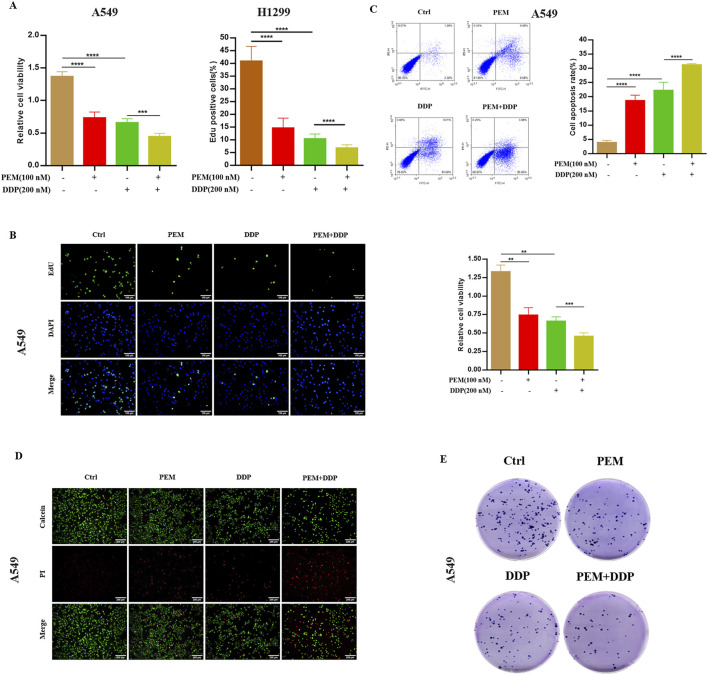
PEM boosts the antitumor effect of DDP *in vitro*. The indicated NSCLC cells were subjected to PEM (100 nM), or DDP (200 nM), or PEM (100 nM) and DDP (200 nM) for 72 h. **(A)** Cell viability was assessed using the CCK-8 assay. **(B)** Cell proliferation was detected by 5-ethynyl-2′-deoxyuridine (EdU) incorporation assay. Representative images and statistical histograms are shown. Scale bar: 100 µm. **(C)** A549 cells were tested by annexin V-FITC/PI assay. **(D)** The A549 cells were subjected to calcein-AM/PI staining (calcein-AM: live cells, PI: dead cells). Scale bar: 100 µm. **(E)** A colony formation assay of A549 cells was performed. Representative images and corresponding quantitative histograms from three independent experiments are shown (^**^ *P* < 0.01; ^***^ *P* < 0.005; ^****^ *P* < 0.001).

First, we treated human NSCLC A549 or H1299 cells with PEM, DDP, or PEM and DDP and tested the cytotoxicity through the CCK-8 assay. The results showed that PEM and DDP both inhibited the growth of cells. In addition, PEM increased the cisplatin-induced cytotoxic effect in cells (Figure 1A). Next, we tested whether the combination treatment could suppress the proliferation of A549 cells. As expected, either PEM or DDP treatment inhibited EdU incorporation into cell nuclei, while PEM co-administration with DDP increased the reduction of the percentage of EdU^+^ cells, suggesting that PEM in combination with DDP significantly disrupted NSCLC cancer growth (Figure 1B). We then utilized the annexin V-FITC/PI assay and calcein-AM/PI staining to further clarify the synergetic effect of PEM and DDP, demonstrating that the combination of PEM boosted the DDP-induced decrease in the number of viable cells and increased the number of dead cells (Figures 1C,D). In addition, we examined the combined effect of PEM and DDP on the capacity of long-term cell viability by the clonogenic survival assay in A549 cells. As expected, cloned clusters of A549 cells exposed to the combinative treatment were fewer and smaller compared with those exposed to either agent alone after culture for 3 days (Figure 1E). Collectively, these results suggested that pemetrexed sensitizes the cisplatin-induced cytotoxic effect in NSCLC cells.

### 3.2 Pemetrexed enhances cisplatin-induced cytotoxic effects by inducing ferroptosis in NSCLC cells

**FIGURE 2:**
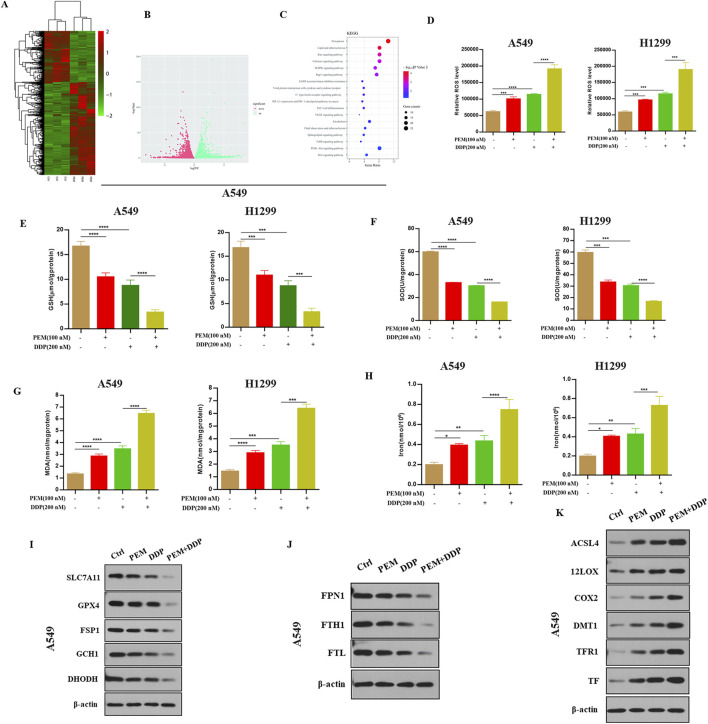
PEM induces ferroptosis in cells. A549 cells were grown with or without PEM (100 nM) for 72 h. **(A)** Heatmap summarizing RNA-seq data for the control and PEM-treated A549 cells with 744 upregulated genes (Red) and 412 downregulated genes (Green). **(B)** Volcano plot of differentially expressed genes between the control and PEM-treated cells as determined by RNA-seq. **(C)** KEGG enrichment analysis of PEM-regulated genes. The A549 or H1299 cells were subjected to PEM (100 nM), or DDP (200 nM), or PEM (100 nM) and DDP (200 nM) for 72 h. **(D)** The intracellular ROS was tested by flow cytometry, and the quantification of fluorescence intensities is shown. The levels of GSH **(E)**, SOD **(F)**, MDA **(G)**, and intercellular iron **(H)** were measured using appropriate kits, and the corresponding statistical results are shown. Western blot was used to detect **(I)** the expression of anti-ferroptosis proteins (SLC7A11, GPX4, DHODH, FSP1, and GCH1) and **(J)** FPN1, FTH1, FTL, and **(K)** the expression of pro-ferroptosis proteins (ACSL4, 12LOX, COX2, DMT1, TFR1, and TF) in A549 cells. (^*^ *P* < 0.01; ^**^ *P* < 0.01; ^***^ *P* < 0.005; ^****^ *P* < 0.001).

Previous studies have shown that cisplatin exerts antitumor action by inducing ferroptosis (Guo et al., 2018), indicating that pemetrexed may enhance cisplatin-induced cytotoxic effect by inducing ferroptosis in NSCLC cells. In this study, we conducted a genome-wide RNA-seq analysis to explore the molecular mechanism underlying pemetrexed-mediated antitumor activity. Total RNAs were isolated and sequenced from A549 cells treated with pemetrexed or control. The pemetrexed challenge results in a total of 1156 genes (744 upregulated and 412 downregulated) being differentially expressed (|fold change| ≥ 2, *P* < 005) (Figures 2A,B). KEGG enrichment analysis found that ferroptosis is the most enriched among several biological pathways (Figure 2C). We observed that PEM boosted the cisplatin-induced increased concentrations of ROS (Figure 2D), decreased levels of GSH (Figure 2E) and SOD (Figure 2F), and increased content of MDA (Figure 2G) and concentrations of iron (Figure 2H) in A549 cells. Western blot analysis revealed that PEM facilitated DDP-mediated downregulation of the expression of anti-ferroptosis proteins (SLC7A11, GPX4, DHODH, FSP1, and GCH1) (Figure 2I) and FPN1, FTH1, and FTL (Figure 2J) and upregulated the expression of pro-ferroptosis proteins (ACSL4, 12LOX, COX2, DMT1, TFR1, and TF) (Figure 2K). These results indicated that PEM enhances cisplatin-induced cytotoxic effect by inducing ferroptosis.

### 3.3 Inhibition of ferroptosis reverses cisplatin-induced ferroptosis in NSCLC cells

**FIGURE 3:**
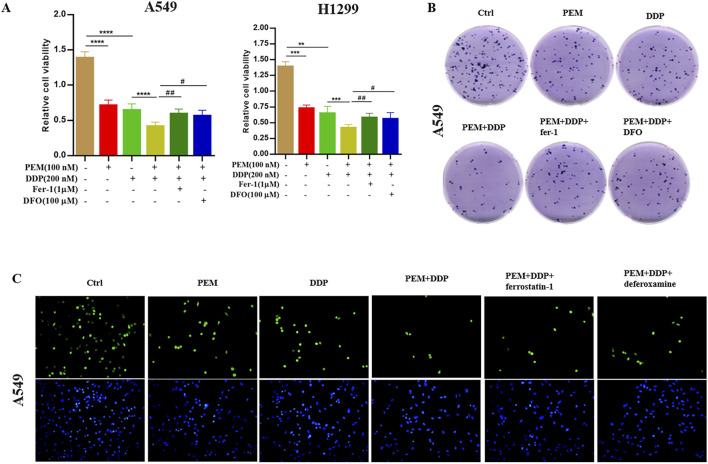
Ferroptosis inhibitors reverse PEM plus DDP-induced suppression of cell proliferation. The NSCLC A549 or H1299 cells were subjected to PEM (100 nM), DDP (200 nM), or PEM (100 nM) and DDP (200 nM) for 72 h after pretreatment with ferroptosis inhibitors for 2 h. **(A)** Cell viability was assessed using the CCK-8 assay in A549 and H1299 cells. **(B)** A colony formation assay of the A549 cells was conducted. **(C)** Cell proliferation was assessed by the EdU incorporation assay. (^**^ *P* < 0.01; ^***^ *P* < 0.005; ^****^ *P* < 0.001; ^#^ *P* < 0.05; ^##^ *P* < 0.01).

**FIGURE 4:**
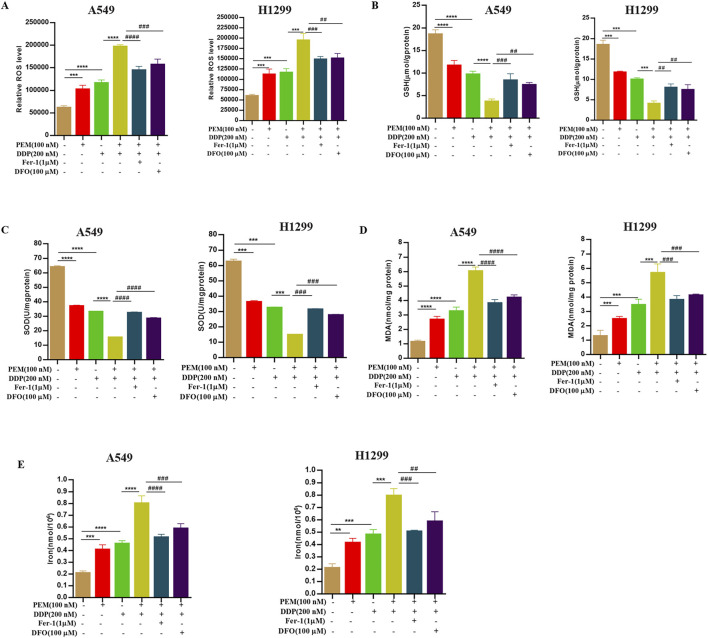
Ferroptosis inhibitors reverse PEM plus DDP-induced ferroptosis in NSCLC cells. The NSCLC A549 and H1299 cells were subjected to PEM (100 nM), DDP (200 nM), or PEM (100 nM) and DDP (200 nM) for 72 h after pretreatment with ferroptosis inhibitors for 2 h. The ROS production **(A)**, levels of GSH **(B)**, SOD **(C)**, MDA **(D)**, and intercellular iron **(E)** were assessed using the appropriate kits, and the corresponding statistical results are shown. (^***^ *P* < 0.005; ^****^ *P* < 0.001; ^##^ *P* < 0.01; ^##^ *P* < 0.01; ^###^ *P* < 0.005; ^####^ *P* < 0.001).

To further confirm that the PEM-induced cytotoxic effect is channeled through inducing ferroptosis in A549 cells, we used the ferroptosis inhibitors deferoxamine (DFO) and ferrostatin-1 (Fer-1) in PEM and DDP-induced cells. DFO and Fer-1 can reverse PEM- and DDP-induced ferroptosis in cells. The rescued phenotypes included alleviated loss of cell viability (Figure 3A), increased numbers of colony formations (Figure 3B), a remarkably reduced percentage of EdU+ cells (Figure 3C), decreased concentrations of ROS (Figure 4A), increased GSH level (Figure 4B), decreased content of SOD (Figure 4C) and MDA (Figure 4D), and decreased iron (Figure 4E) in A549 cells. These results suggested that PEM enhances cisplatin-induced cytotoxic effect by inducing ferroptosis.

### 3.4 Pemetrexed promotes cisplatin-induced ferroptosis by the SLC7A11/GPX4 axis in NSCLC cells

**FIGURE 5:**
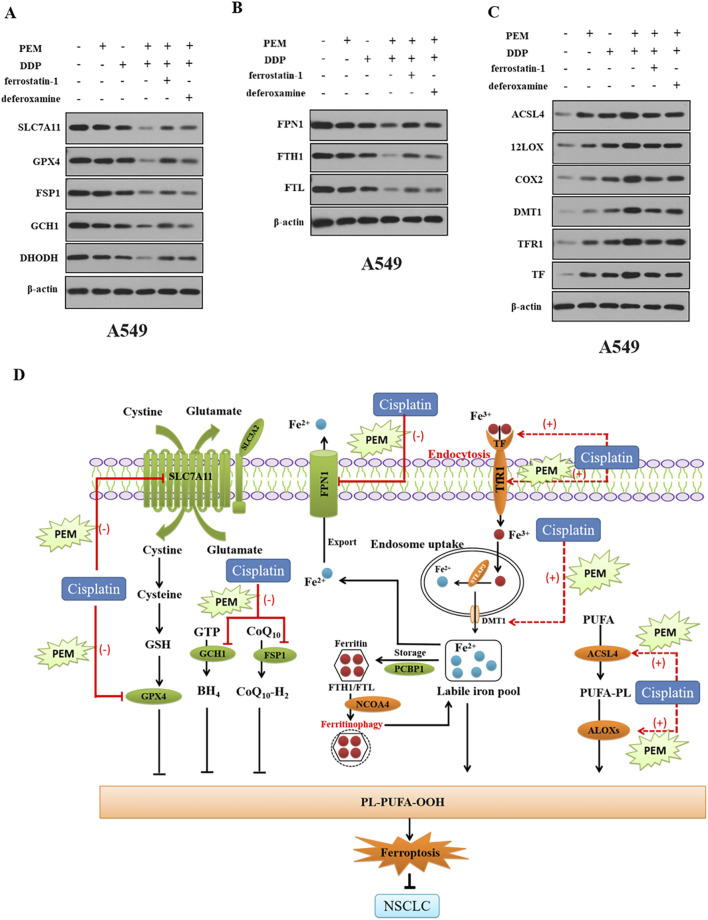
Pemetrexed modulates SLC7A11/GPX4, ACSL4, and iron. The NSCLC A549 cells were subjected to PEM (100 nM), DDP (100 nM), or PEM (100 nM) and DDP (200 nM) for 72 h. Western blot was used to detect **(A)** SLC7A11/GPX4, **(B)** ACSL4 and 12LOX, and **(C)** the expression of FPN1, FTH1, FTL, DMT1, and TfR1 in A549 cells. The NSCLC A549 cells were subjected to PEM (100 nM), DDP (200 nM), or PEM (100 nM) and DDP (200 nM) for 72 h with and without pretreatment with ferroptosis inhibitors for 2 h. Western blot was used to detect **(D)** SLC7A11/GPX4, **(E)** ACSL4 and 12LOX, and **(F)** the expression of FPN1, FTH1, FTL, DMT1, and TfR1 in A549 cells.

Next, we examined the underlying mechanism by which PEM induces ferroptosis in A549 cells. Western blot analysis uncovered that PEM and DDP downregulate the expression of SLC7A11, GPX4, FPN1, FTH1, FTL, DHODH, FSP1, and GCH1 in A549 cells (Figures 5A,B) and upregulate the expression of ACSL4, 12LOX, COX2, DMT1, TFR1, and TF (Figure 5C). However, the effects were reversed by ferroptosis inhibitors ferrostatin-1 or deferoxamine in NSCLC cells. DFO or Fer-1 can reverse PEM-plus-DDP-induced altered expression of anti-ferroptosis proteins and pro-ferroptosis proteins (Figures 5A–C). These results suggest PEM inhibits the SLC7A11/GPX4 system, enhances lipid metabolism, and modulates the accumulation of iron.

## 4 Discussion

Chemotherapy resistance (both intrinsic and acquired) often occurs during treatment and leads to the poor prognosis of patients with NSCLC. Ferroptosis is a novel form of regulated cell death that is morphologically, biochemically, and genetically different from other types of cell death (Wang et al., 2023a). Mounting evidence has suggested that pharmacologically inducing ferroptosis might be a promising strategy to kill even cancer cells with resistance to chemotherapy (Wang et al., 2023b). In the present study, we for the first time reveal that pemetrexed boosts the antitumor activity of cisplatin by inducing ferroptosis in NSCLC cells. Mechanistic insights revealed that pemetrexed may downregulate SLC7A11/GPX4, upregulate ACSL4, and promote cellular iron uptake (TfR1), while decreasing iron storage (FTH-1 and FTL) and iron output (PFN1) in NSCLC cells. These findings provide novel insight into killing cancer by pemetrexed through inducing ferroptosis, highlighting a novel potential target for the treatment of NSCLC (Figure 5D).

Systemic platinum-based chemotherapy is still recommended as the standard first-line regimen for patients with advanced and earlier-stage NSCLC (Nagasaka and Gadgeel, 2018). Platinum-based chemotherapy combined with pemetrexed is frequently recommended as one of the first-line therapeutic regimens for NSCLC. However, chemoresistance to platinum poses a significant challenge in the treatment of NSCLC individuals (Yin et al., 2016; Wang et al., 2024). Therefore, novel mechanisms are needed to treat NSCLC and meet unmet needs in NSCLC treatment (Herbst et al., 2018; Lim and Ma, 2019). During the past decade, researchers have found that ferroptosis can overcome drug resistance and increase the death of tumor cells (Wang et al., 2023). Emerging evidence suggests that agents with ferroptosis-inducing activity increase the chemosensitivity of classic therapeutic agents by triggering ferroptosis (Huang et al., 2024). In the present study, whether pemetrexed increases sensitivity to cisplatin via ferroptosis remains to be determined.

Recently, both GPX4-dependent and GPX4-independent pathways with distinct subcellular localizations have been identified as critical regulators of ferroptosis, which include the GPX4/GSH axis (Lei et al., 2022; Sun et al., 2022), the FSP1/CoQH_2_ system (Bersuker et al., 2019; Doll et al., 2019; Nakamura et al., 2023), the GCH1/BH_4_ system (Kraft et al., 2020; Soula et al., 2020), and the DHODH/CoQH_2_ system (Mao et al., 2021). The inhibition of the antioxidant SLC7A11/GSH/GPX4 system and the accumulation of free iron are two key signals that induce ferroptosis (Chen et al., 2021). The SLC7A11/GSH/GPX4 system is the main ferroptosis defense system. Our results showed a decrease in expression protein levels of SLC7A11/GPX4, FSP1, GCH1, and DHODH after PEM treatment, and PEM boosts the DDP-mediated downregulation of these proteins, suggesting that PEM induces ferroptosis by inactivating various inhibitors of ferroptosis and thereby increasing the sensitivity of NSCLC cells to cisplatin. The present findings substantiate previous indications that PEM plus DDP-induced ferroptosis in NSCLC (Bi et al., 2022).

Functioning as a fatty acid metabolism enzyme, acyl-coenzyme A [CoA] synthetase long-chain family member 4 (ACSL4) is a driver of ferroptosis. ACSL4 ligates polyunsaturated fatty acids (PUFAs) with CoA to produce acyl-CoA. ACSL4 increases the content of polyunsaturated fatty acids (PUFAs) in phospholipids (Doll et al., 2017). Once polyunsaturated acyl tails (PL-PUFAs) are incorporated into membrane environments, lipoxygenases (LOXs), cytochrome P450 oxidoreductase (POR), and labile iron use molecular oxygen (O_2_) to do a peroxidation reaction, leading to the generation of peroxidated PL-PUFAs (PL-PUFA-OOH), thereby inducing ferroptosis (Hadian and Stockwell, 2020; Zou et al., 2020). Our results showed increased protein levels of ACSL4 and 12LOX after PEM treatment. PEM boosts the DDP-mediated upregulation of ACSL4 and 12LOX, indicating that PEM induces ferroptosis by activating ACSL4 and 12LOX and thereby enhancing the sensitivity of NSCLC cells to cisplatin.

Tumor cells are more susceptible to ferroptosis because they have higher iron requirements than normal cells (Hassannia et al., 2019). Iron metabolism contributes to ferroptosis. The iron (Fe^3+^) binds to transferrin (Tf) in the circulation to form a complex holotransferrin (holo-Tf) (Ganz, 2008), which binds to transferrin receptor 1 (TfR1) and then enters the cells through clathrin-mediated endocytosis (McCarthy and Kosman, 2012). Then, the Fe^3+^–Tf–TfR complex is transported into cells to the endosome, where Fe^3+^ detaches from Tf and is reduced to Fe^2+^ by duodenal cytochrome b (DCYTB) (Tulpule et al., 2010) or the metalloreductase six-transmembrane epithelial antigen of prostate 3 (STEAP3) (Ohgami et al., 2005). Then, divalent metal transporter 1 (DMT-1) mediates Fe^2+^ entering the cytosol (De Domenico et al., 2008). The unbound Fe^2+^ can be stored in a non-toxic form as Fe^3+^ by ferritin, which consists of two isoforms, that is, heavy (FTH) and light (FTL). PEM plus DDP downregulate the expression of FPN1, FTH1, and FTL and upregulate the expression of DMT1 and TfR1 in A549 cells. These results suggest that PEM promotes cellular iron uptake and decreases iron storage and iron output.

In summary, the present study uncovers a novel mechanism underlying how PEM kills NSCLC cells *in vitro* by inducing ferroptosis through inactivating various inhibitors of ferroptosis, activating ACSL4 and 12LOX, promoting cellular iron uptake, and decreasing iron storage and iron output.

While our findings are derived from *in vitro* models, they offer several translational implications for NSCLC therapy. First, the synergistic induction of ferroptosis by PEM and DDP provides a mechanistic rationale for optimizing this clinically established combination. Monitoring ferroptosis-related biomarkers, such as lipid peroxidation products, GPX4 activity, or iron metabolism proteins, in patients’ tumors could help identify subsets more likely to respond to PEM/DDP regimens. Second, our data suggest that tumors with inherent or acquired resistance to cisplatin might be re-sensitized through ferroptosis induction. This supports the exploration of pharmacological ferroptosis inducers (e.g., GPX4 inhibitors or iron-loading agents) as adjuncts to platinum-based chemotherapy in refractory NSCLC. Finally, the involvement of multiple ferroptosis defense pathways (SLC7A11/GPX4, FSP1, DHODH, and GCH1) implies that tumor heterogeneity in ferroptosis vulnerability may influence therapeutic outcomes. Profiling these pathways in clinical samples could guide patient stratification and personalized combination strategies. Future studies validating these mechanisms in *in vivo* models and correlating them with clinical response data will be essential to translate these insights into improved therapeutic paradigms for NSCLC.

## Data Availability

The data presented in the study are deposited in the figshare repository, this data can be found here: https://doi.org/10.6084/m9.figshare.30945029; https://doi.org/10.6084/m9.figshare.30945077.
