# Supplementary material for: Pemetrexed sensitizes cisplatin therapy by inducing ferroptosis in NSCLC cells
Source: Front Pharmacol. 2026 Jan 21;16:1764937. doi: 10.3389/fphar.2025.1764937 (PMC12868191; doi:10.3389/fphar.2025.1764937)
Supplement: Supplementary file 1 [file Table1.doc]

**KEY RESOURCES TABLE**

| **REAGENT or RESOURCE** | **SOURCE** | **IDENTIFIER** | **Dilution** |
| --- | --- | --- | --- |
| GPX4 antibody | abclonal | A1933 | 1:500 |
| SLC7A11 antibody | affinity | DF12509 | 1:500 |
| FSP1 antibody | affinity | DF6516 | 1:1000 |
| GCH1 antibody | affinity | DF9546 | 1:400 |
| DHODH antibody | proteintech | 14877-1-AP | 1:500 |
| ACSL4 antibody | Abclonal | A6826 | 1:500 |
| 12LOX antibody | santa | sc-365194 | 1:300 |
| COX2 antibody | wanleibio | WL01750 | 1:400 |
| DMT1 antibody | wanleibio | WL05401 | 1:300 |
| TFR1 antibody | wanleibio | WL03500 | 1:500 |
| TF antibody | wanleibio | WL01221 | 1:1000 |
| FPN1 antibody | affinity | DF13561 | 1:500 |
| FTH1 antibody | wanleibio | WL05360 | 1:500 |
| FTL antibody | affinity | DF6604 | 1:1000 |
| β-actin antibody | wanleibio | WL01372 | 1:1000 |
| DDP | meilunbio | MB1055 | - |
| PEM | MACKLIN | P859885 | - |
| ferrostatin-1 | MACKLIN | F864515 | - |
| deferoxamine | MACKLIN | D873692 | - |
| BCA Kit | wanleibio | WLA004 | - |
| CCK-8 Kit | wanleibio | WLA074 | - |
| ROS Kit | wanleibio | WLA131 | - |
| Wright-Giemsa stain solution | KeyGEN | KGE1107 | - |
| Cell apoptosis assay kit | wanleibio | WLA001 | - |
| EdU Kit | KeyGEN | KGA9602 | - |
| GSH Kit | wanleibio | WLA105 | - |
| MDA Kit | wanleibio | WLA048 | - |
| SOD Kit | wanleibio | WLA110 | - |
| Iron Kit | Elabscience | E-BC-K881-M | - |
