# Supplementary figures and images for: Pemetrexed sensitizes cisplatin therapy by inducing ferroptosis in NSCLC cells
Source: Front Pharmacol. 2026 Jan 21;16:1764937. doi: 10.3389/fphar.2025.1764937 (PMC12868191; doi:10.3389/fphar.2025.1764937)

**Fig 2F**


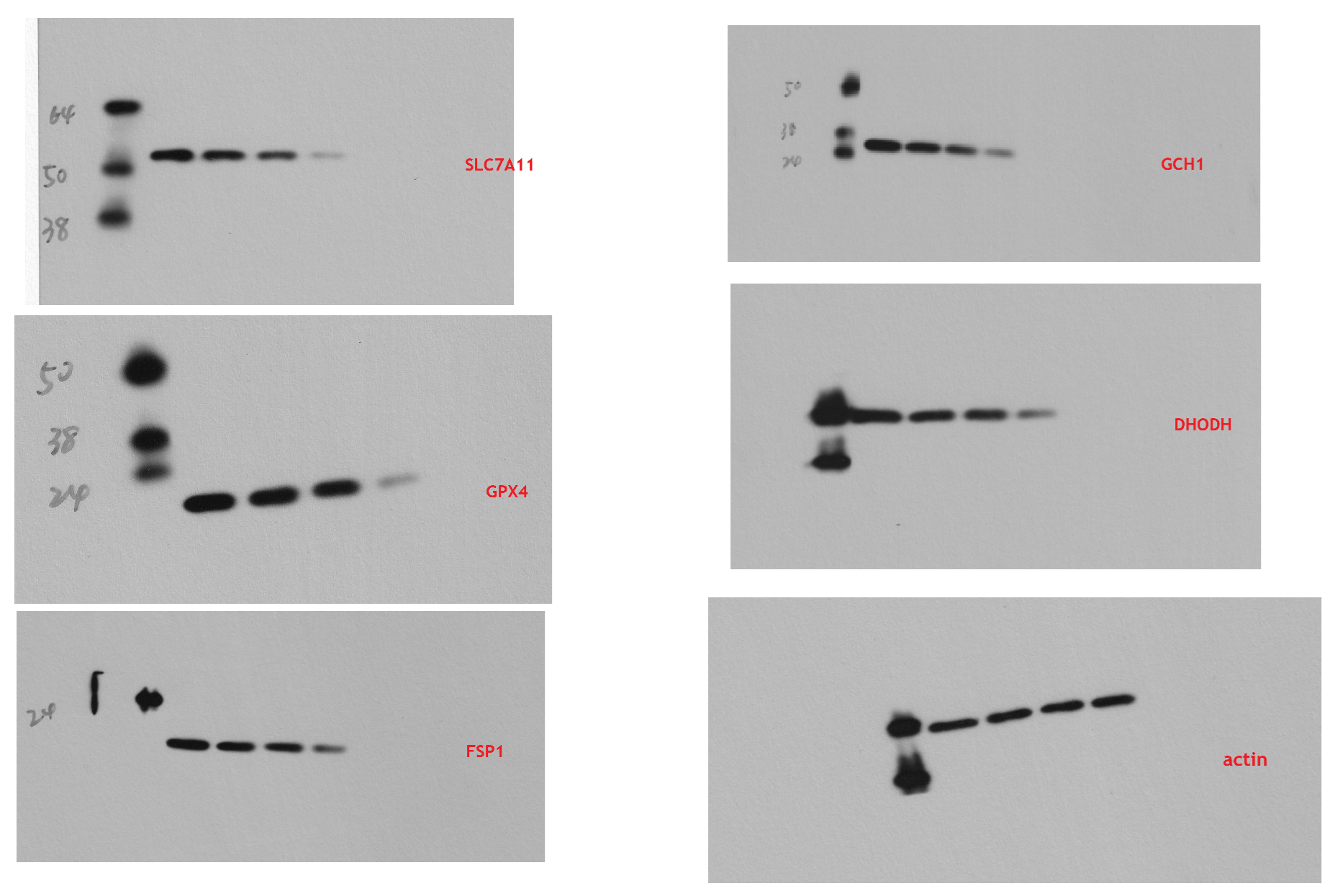


**Fig 2G**


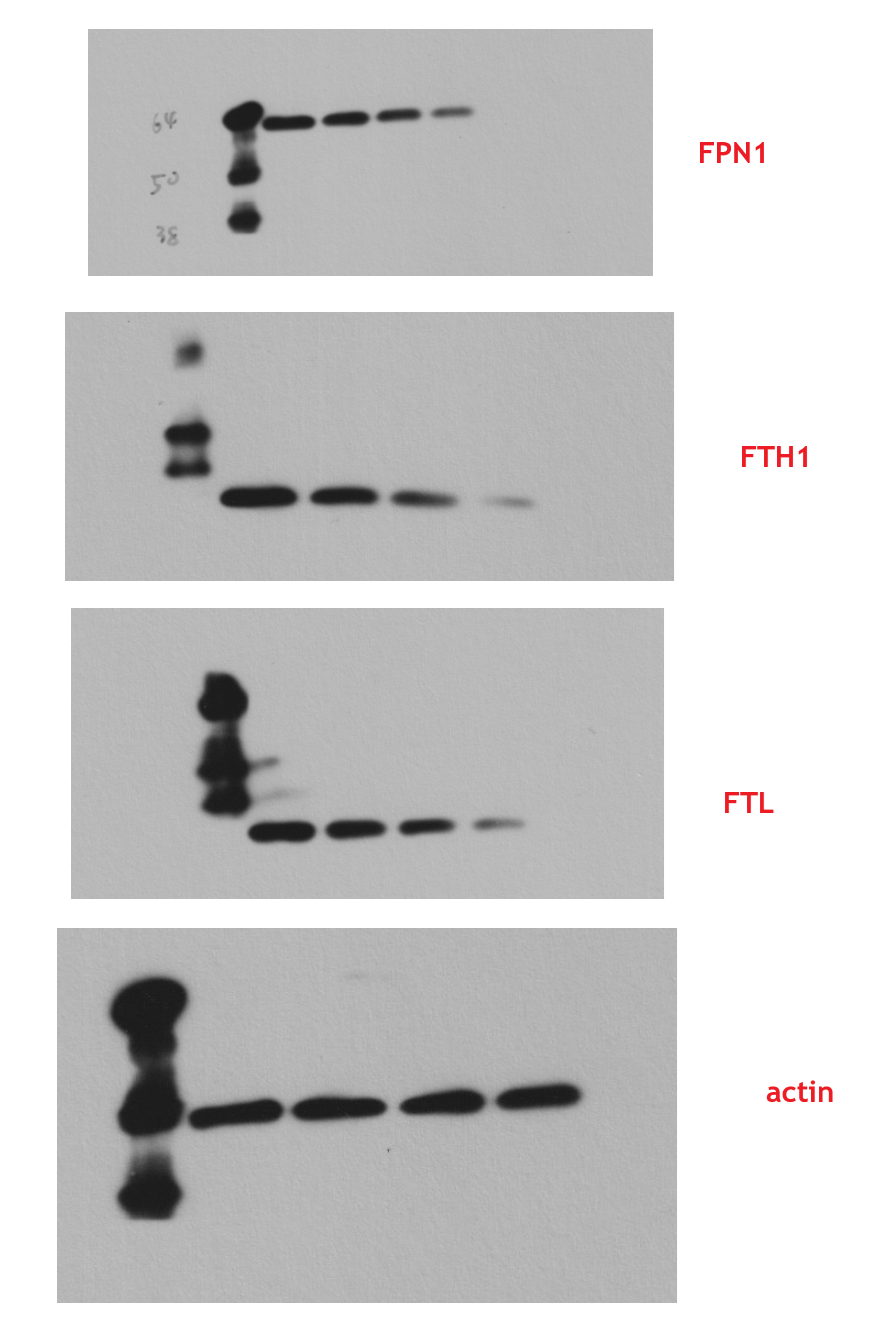


**Fig 2H**


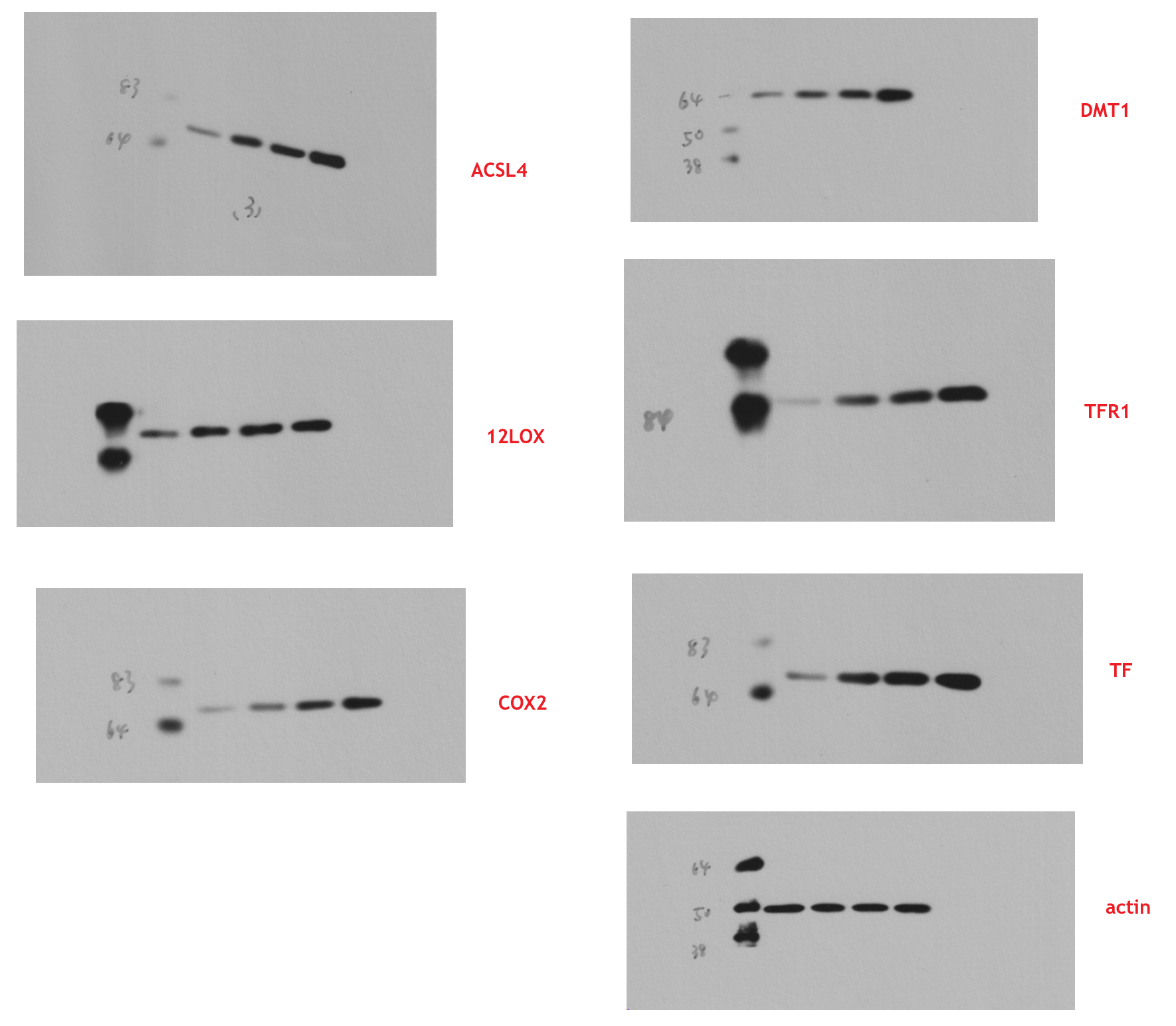


**Fig 5A**


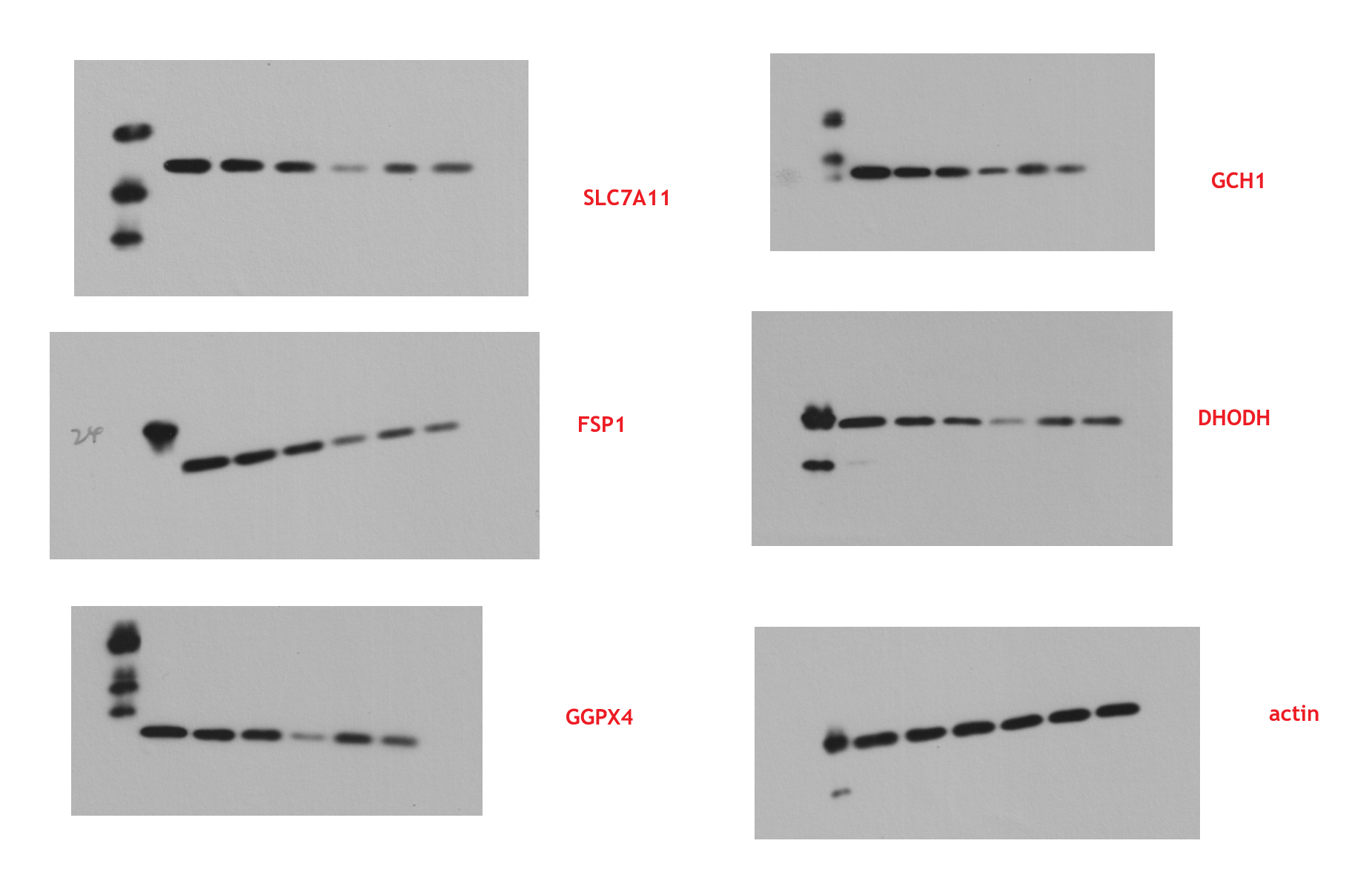


**Fig 5B**


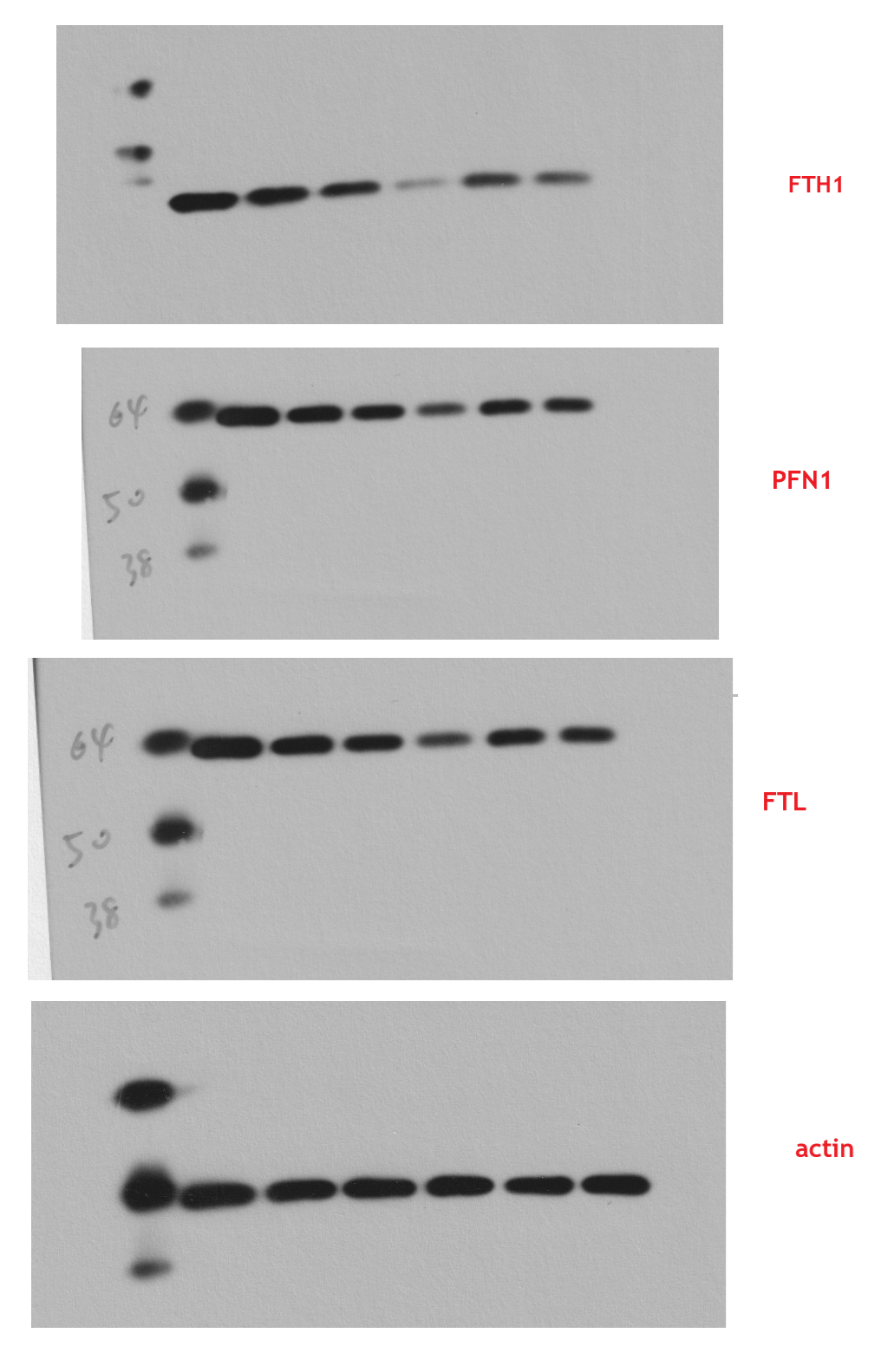


**Fig 5C**


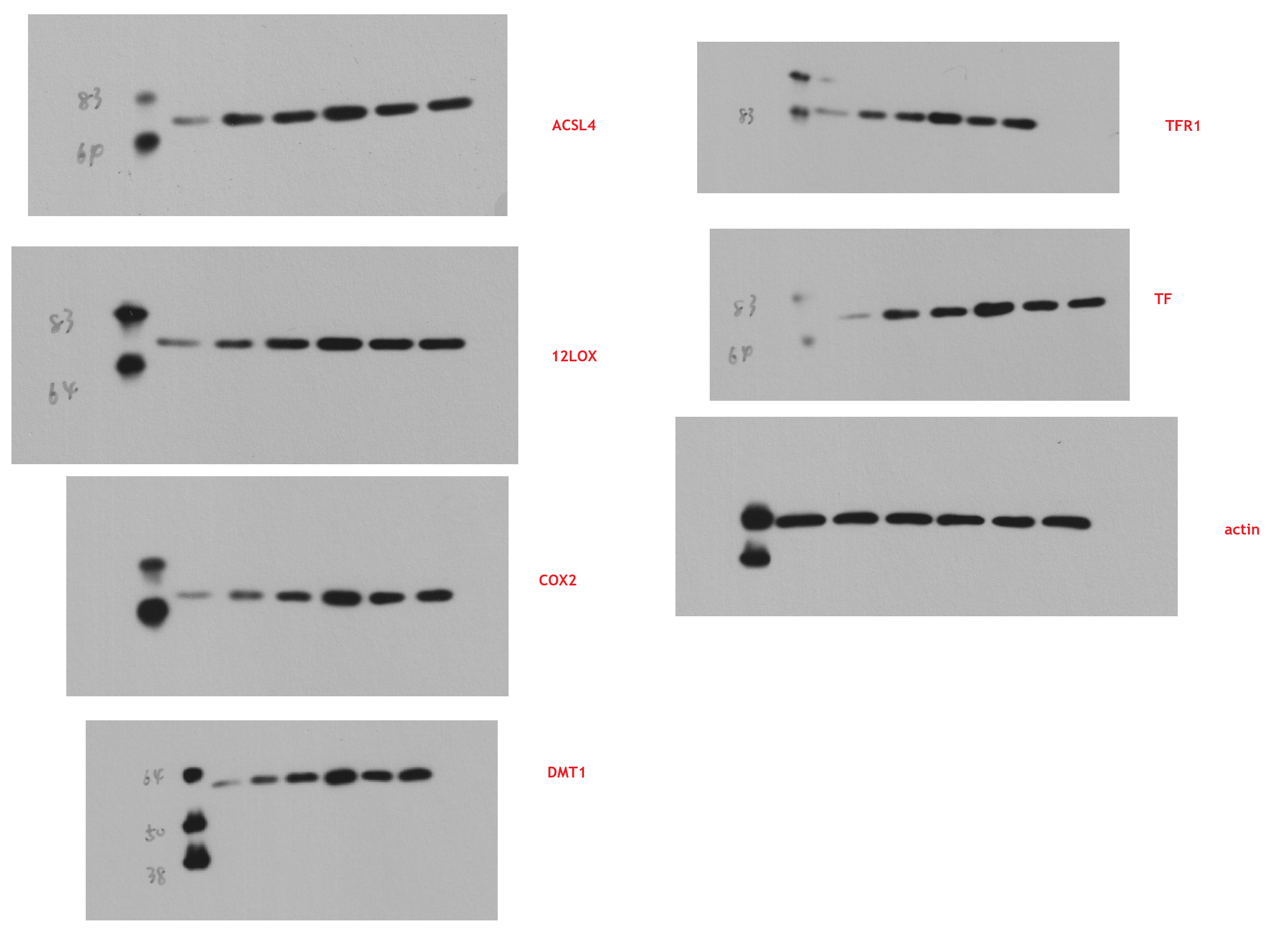

Supplement: Supplementary file 2 [file Supplementaryfile1.doc]
